# Recent Therapies and Biomarkers in Mucinous Ovarian Carcinoma

**DOI:** 10.3390/cells14161232

**Published:** 2025-08-09

**Authors:** Grzegorz Przywara, Oliwia Biegańska, Emilia Biczak, Aleksander Białoń, Dominik Fidorowicz, Alicja Dankowska, Zofia Łapińska, Julita Kulbacka

**Affiliations:** 1 Faculty of Medicine, Wroclaw Medical University, Mikulicza-Radeckiego 5, 50-345 Wroclaw, Poland; grzegorz.przywara@student.umw.edu.pl (G.P.); oliwia.bieganska@student.umw.edu.pl (O.B.); emilia.biczak@student.umw.edu.pl (E.B.); aleksander.bialon@student.umw.edu.pl (A.B.); dominik.fidorowicz@student.umw.edu.pl (D.F.); alicja.dankowska@student.umw.edu.pl (A.D.); 2 Student Research Group No. K148, Faculty of Pharmacy, Wroclaw Medical University, Borowska 211A, 50-556 Wroclaw, Poland; 3 Department of Molecular and Cellular Biology, Wroclaw Medical University, Borowska 211A, 50-556 Wroclaw, Poland; zofia.lapinska@umw.edu.pl; 4 Department of Immunology and Bioelectrochemistry, State Research Institute Centre for Innovative Medicine, LT-08406 Vilnius, Lithuania

**Keywords:** mucinous ovarian carcinoma, targeted therapy, HER2, KRAS, immunotherapy, checkpoint inhibitors, synthetic lethality, PARP inhibitors, Wnt/β-catenin pathway

## Abstract

Mucinous ovarian carcinoma (MOC) represents a rare and biologically distinct subtype of ovarian cancer, characterized by poor response to standard platinum-based chemotherapy and a unique molecular profile, including frequent KRAS mutations and HER2 amplifications. Recent advancements in targeted therapy, such as HER2 inhibitors and *KRAS^G12C^* inhibitors, offer promising avenues for personalized treatment. Immunotherapy, particularly checkpoint inhibitors, shows potential in tumors with high PD-L1 expression or tumor mutational burden. Novel strategies, including antibody–drug conjugates, synthetic lethality approaches, and Wnt/β-catenin pathway inhibitors, are reshaping the therapeutic landscape. Despite these developments, challenges such as intratumoral heterogeneity and therapy resistance persist, underscoring the need for innovative clinical trial designs and combination regimens. This review synthesizes the latest advancements in MOC therapies, highlighting opportunities for improved outcomes in this challenging malignancy.

## 1. Introduction

Cancer remains a leading cause of morbidity and mortality worldwide, with ovarian cancer representing the most lethal gynecological malignancy due to its often late diagnosis and complex biology. Advances in cancer treatment have evolved from surgery and cytotoxic chemotherapy to include targeted therapies and immunomodulatory approaches, reflecting a deeper understanding of tumor heterogeneity and molecular drivers [1]. Despite these advances, ovarian cancer is not a single disease but comprises several histological subtypes, each with distinct clinical presentations, molecular features, and responses to therapy.

Epithelial ovarian carcinomas (EOCs) account for around 90% of these cases, with mucinous ovarian carcinoma (MOC) representing 3% to 4% of all EOC diagnoses [2,3]. Its prevalence may be overestimated because some cancers classified as MOCs are actually metastatic. One study showed that of 52 diagnosed MOCs, only 23% were primary cancers; the rest were metastatic [4]. In another study, 71% of MOCs were metastatic [3].

Clinically, MOC often presents with non-specific symptoms such as abdominal distension or pain, which can overlap with other ovarian tumors but may be less likely to cause the rapid progression and ascites typical of serous tumors. Biomarker profiles further distinguish these subtypes: while CA125 is elevated in the vast majority of serous carcinomas (up to 99%), its sensitivity is notably lower in mucinous tumors, with only about 69% showing abnormal levels and generally at lower absolute values [5,6,7,8]. This reduced sensitivity of CA125 in MOC contributes to diagnostic challenges and underscores the need for additional biomarkers, such as MUC13, which may improve the detection of non-serous subtypes [9].

When comparing MOC with serous ovarian carcinoma (SOC), worse treatment outcomes and higher mortality have been demonstrated in advanced MOC [3,5,10]. The reason for the poor prognosis may be resistance to chemotherapy (CT) [11]. In MOC, numerous mutations occur, including *KRAS*, *TP53*, *CDKN2A*, *PIK3CA*, *PTEN*, *BRAF*, *FGFR2*, *STK11*, *CTNNB1*, *SRC*, *SMAD4*, *GNA11*, and *ERBB2* (HER2), with *KRAS* mutation being the most common (ranging from 43.6% to 64.9% depending on the study) [12,13,14]. Additionally, the *KRAS* mutation is considerably more common in MOCs compared to other ovarian cancer types [15]. The co-occurrence of *KRAS* mutation with *ERBB2* amplification, as well as *BRAF* and *TP53* mutations, has been observed [12,13]. It has also been demonstrated that the presence of the *KRAS* mutation in MOC correlates with platinum resistance and is the strongest predictor of definite malignancy and poor prognosis [15,16,17]. The *TP53* mutation occurs in ~48.9–57% of MOCs and is associated with high-grade cancer, advanced FIGO stage, intraoperative residual disease > 1 cm, and poor overall survival [12,13,18].

Overexpression of HER2 amplification occurs in ~18.8% of MOCs [14]. Interestingly, this rate is significantly higher in the Asian population (27.4–33.3%) [19,20]. Considering the spectrum of mutations, targeted therapy for MOCs appears promising.

**Figure 1:**
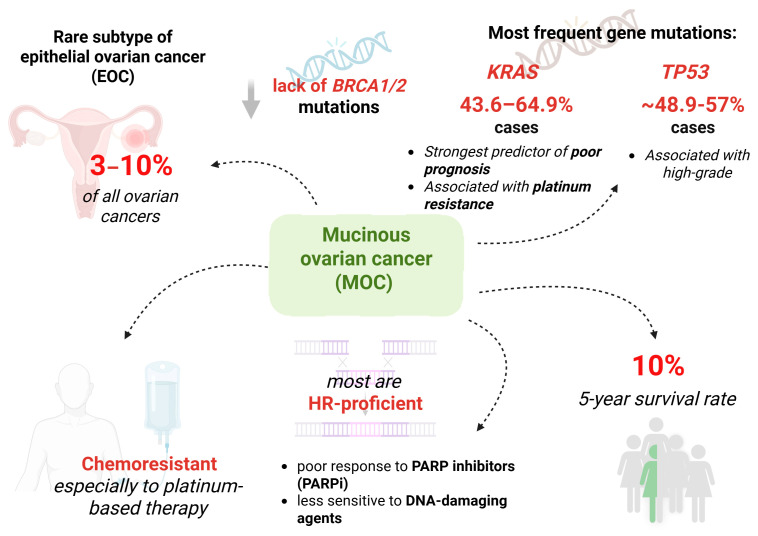
Key molecular and clinical features of mucinous ovarian cancer (MOC); 5-year survival rate—a statistical measure used in medicine to indicate the percentage of people who are still alive five years after being diagnosed with a particular disease; HR—low homologous recombinationCreated in BioRender. Łapińska, Z. (2025) https://BioRender.com/6fbh3ik.

MOC is characterized by the lack of *BRCA* mutations and low homologous recombination (HR) deficiency compared to SOC. A relatively high presence of PD-L1 expression is present in approximately 14% of cases [21,22]. Other actionable targets identified through multi-omics studies include *CDK1*, *CDC20*, *PRC1*, *CCNA2,* and *TRIP13* [23]. Figure 1 summarizes the prevalence, genetic alterations, treatment response, and therapeutic targets in mucinous ovarian cancer.

Differences in presentation and biomarker expression highlight an unmet clinical need for subtype-specific research and tailored therapeutic strategies. A nuanced understanding of MOC’s distinct biology and clinical behavior is essential for improving early detection, prognostication, and treatment outcomes, justifying focused reviews and research efforts in this area.

## 2. Materials and Methods

This scoping review was conducted in accordance with the methodological framework proposed by Arksey et al. [24], which includes five key stages: identifying the research question, identifying relevant studies, study selection, charting the data, and collating, summarizing, and reporting the results. The review process was further guided by the PRISMA-ScR (Preferred Reporting Items for Systematic reviews and Meta-Analyses extension for Scoping Reviews) checklist to ensure methodological transparency and comprehensive reporting [25].

Original open-access articles in English describing new and potential therapies in mucinous ovarian carcinoma (MOC) were sought using the search term “ovarian mucinous carcinoma” in combination with the following keywords: “treatment”, “chemotherapy”, “surgery”, “HER2-targeted therapy”, “KRAS inhibitors”, “Src pathway”, “immunotherapy”, “adoptive cell therapy”, “checkpoint inhibitors”, “hormonal therapy”, “synthetic lethality”, “PARP inhibitors”, “ATR inhibitors”, and “Wnt/β-catenin pathway”. The databases PubMed, Google Scholar, and ScienceDirect were searched. Publications published between 1996 and 2025 were considered eligible.

## 3. Standard Treatments

The basis of ovarian cancer therapy is combined treatment. It involves the use of surgical methods and chemotherapy (CT) [18].

### 3.1. Surgical Treatment

The surgery aims to confirm the diagnosis, determine the stage of the cancer, and perform a complete cytoreduction of the tumor, with optimal efficiency. Depending on the clinical stage, the scope of the procedure and complementary therapy are determined. It is largely similar to the surgical management of other forms of epithelial ovarian cancer (EOC) [26]. The primary surgical treatment includes bilateral salpingo-oophorectomy, total hysterectomy, omentectomy, and removal of any visible tumor metastases. Random biopsies of the peritoneum for cytological examinations, as well as pelvic washings, should also be conducted. Diagnosis at an early stage, with surgical resection of large unilateral tumors, results in well-curative outcomes in many cases [18]. Evaluation of retroperitoneal lymph nodes is essential for staging surgery in all types of EOCs. However, in early-stage MOC, its role is debatable [27,28]. The role of appendectomy is also controversial. Due to older sources, it is recommended in all mucinous ovarian tumors but is usually applied only when the appendix is abnormal [29].

In young women (under 40 years old) who wish to preserve fertility and meet the criteria of cancer confined to one ovary without capsule invasion and intraperitoneal adhesions, fertility-sparing surgery can be performed. This therapeutic solution involves the removal of the affected ovary along with the fallopian tube while preserving the uterus and the other ovary. Such an approach, apart from addressing reproductive concerns, also prevents the occurrence of diseases such as osteoporosis and coronary disease, which are complications of surgical menopause [30,31].

If complete lesion removal is unachievable, optimal cytoreduction is the goal. When this cannot be reached, neoadjuvant CT is recommended. Interval debulking surgery involves operating after three courses of cytostatic treatment, followed by additional therapies, and is advised for patients showing an objective response [26,32]. If the disease progresses after first-line CT, secondary cytoreductive surgery may be considered, although its benefit remains unconfirmed in randomized trials [26].

### 3.2. Chemotherapy (CT)

There are various recommendations for adjuvant chemotherapy treatment of MOCs. The standard approach usually involves platinum-based agents (e.g., carboplatin) and taxanes (such as paclitaxel) [33]. Unfortunately, MOC is often resistant to conventional platinum/taxane-based therapy. The reason is complex, including mechanisms such as multidrug resistance (MDR), low rate of *BRCA1/2* mutations, overexpression of glucose-6-phosphate dehydrogenase (G6PD), Nrf2 pathway activation, apoptosis-related protein inhibition, and others [34]. MDR is the primary reason for treatment failure, resulting from the excessive production of two key membrane proteins: P-glycoprotein (P-gp) and multidrug resistance-associated protein (MRP). *BRCA* mutations improve response to platinum therapy by increasing sensitivity to DNA-damaging treatments such as platinum-based therapies. Patients with these mutations tend to have a better overall response to platinum treatment, which correlates with a longer survival rate in ovarian cancer [34].

Hyperthermic Intraperitoneal Chemotherapy (HIPEC) delivers heated chemotherapy directly into the abdominal cavity during surgery to eliminate residual disease [32]. A study on secondary cytoreductive surgery with HIPEC for recurrent primary MOC showed promising results, with complete, durable responses in two patients and no recurrence at 21 and 27 months. Further research is needed to define its role in ovarian cancer therapy [32].

## 4. Novel Therapeutic Approaches

### 4.1. Molecularly Targeted Treatment Strategies

#### 4.1.1. HER2-Targeted Therapies

Antibody–drug conjugates (ADCs) combine chemotherapy and targeted therapy to treat cancerous tumors. The ADC landscape in OC therapeutics is swiftly evolving, promising more effective treatment methods. Trastuzumab deruxtecan (T-DXd) is a novel HER2-directed ADC that consists of trastuzumab. This humanized monoclonal antibody targets the extracellular domain of HER2 cell surface receptors, a topoisomerase I inhibitor, and a linker protein [35]. It has been demonstrated that T-DXd binds to HER2-positive cells, then DXd acts on the surrounding cells independent of HER2 status. This suggests that the use of trastuzumab deruxtecan increases the efficacy of treatment for HER2-heterogeneous cancers resistant to conventional anti-HER2 therapy [35].

Ten patients with HER2-overexpressing tumors were enrolled in a clinical trial (NCT04482309) with trastuzumab deruxtecan. Among the OC patients, only one patient with the mucinous subtype demonstrated a response to the proposed therapy [36]. This therapeutic solution has shown promising activity in a heavily pretreated cohort of patients with HER2-expressing solid tumors, including ovarian cancer. While the study did not focus only on MOCs, the overall response rate of 37.1% and particularly the improved outcomes in the HER2 3+ subgroup suggest that T-DXd needs further investigation as a potential treatment option for patients with HER2-expressing MOCs, especially after progression on standard therapies [37]. Preclinical studies demonstrate that trastuzumab deruxtecan is effective against HER2-overexpressing high-grade serous (HGSOCs) and clear-cell ovarian cancers (CCOCs), both in vitro and in vivo. The study highlights the potential of HER2-targeted ADCs in treating ovarian cancers with HER2 overexpression [38].

Clinical trials are underway investigating the use of trastuzumab deruxtecan in the treatment of ovarian cancer (NCT04639219, NCT04482309, NCT05417594, NCT05824975, NCT04644068). A phase II clinical trial, BOUQUET, is investigating the use of trastuzumab emtansine in the treatment of persistent or recurrent rare epithelial ovarian tumors, including MOC (NCT04931342). Preclinical studies suggest that another ADC, trastuzumab duocarmazine (SYD985), may also be effective against ovarian cancer, showing stronger anti-cancer activity than trastuzumab emtansine even without high HER2 levels. This effect is likely due to its more potent toxin and its ability to kill nearby cancer cells, which suggests it could be useful against tumors with mixed HER2 expression [39]. Tisotumab vedotin is another conjugate, which is a combination of tisotumab, a monoclonal antibody against tissue factor, and monomethyl auristatin E. Tisotumab vedotin was evaluated in a phase II trial for platinum-resistant ovarian cancer (innovaTV 208-NCT03657043) [40].

The efficacy of Samfenet^®^, a trastuzumab biosimilar, in combination with natural killer (NK) cells was evaluated in a preclinical model of HER2-overexpressing OC. Enhanced sensitivity to Samfenet^®^ was observed in cells exhibiting high HER2 expression compared to those with low HER2 expression. Moreover, the combination of Samfenet^®^ and NK cells significantly enhanced this sensitivity in HER2-high cells and significantly shrank tumors in patient-derived xenograft mouse models, increasing the efficacy observed with Samfenet^®^ monotherapy [41]. The number of studies on the use of HER2-targeted therapy for MOC is insufficient; however, many clinical cases have reported significant improvement after this treatment [40,42,43,44,45]. A case was reported of a patient with *ERBB2*-amplified recurrent MOC who had a poor response to platinum- and taxane-based chemotherapy. The optimal cytoreduction was followed by six cycles of carboplatin–paclitaxel–trastuzumab therapy and one year of trastuzumab maintenance. The patient has remained relapse-free for three years [42]. However, resistance to trastuzumab is also common. In one study, was described the case of a patient with recurrent MOC who developed resistance to trastuzumab–pertuzumab therapy. After treatment with bevacizumab and pyrotinib, the patient achieved remission for 22 months, suggesting pyrotinib as a potential treatment for HER2-positive cancers [43].

#### 4.1.2. Direct *KRAS^G12C^* Inhibitors

*KRAS* mutations are frequently observed in MOCs, highlighting the potential therapeutic value of targeting this pathway [12,13,14]. Clinical investigation of *KRAS* inhibitors in MOCs is currently lacking; the prevalence of these mutations warrants further exploration of this therapeutic strategy. Direct *KRAS* inhibition, especially of the *G12C* mutant (*KRAS^G12C^*), shows promise as a therapeutic strategy [46]. Recent studies reveal that inhibitors like ARS853 not only block the active, GTP-bound form but also engage the inactive GDP-bound conformation by preventing nucleotide exchange, trapping *KRAS* in its inactive state and further suppressing its oncogenic activity. This expanded understanding guides the development of more effective targeted therapies against *KRAS*-driven cancers [47]. The recommended phase II dose of adagrasib was 600 mg twice daily. In patients with *KRAS^G12C^* mutant non-small-cell lung cancer, this dose submitted a confirmed partial response rate of 53.3% with a median duration of response of 16.4 months and a median progression-free survival of 11.1 months [48]. While limited activity was observed in other tumor types, the manageable safety profile, primarily characterized by gastrointestinal adverse events and fatigue, supports further investigation of adagrasib in *KRAS^G12C^*-driven cancers [48]. Clinical trials on the use of direct *KRAS^G12C^* inhibitors are ongoing, but unfortunately, none of them include patients diagnosed with MOC. Trials KRYSTAL-10 (NCT04793958) and CodeBreak300 (NCT05198934) are investigating treatments targeting *KRAS^G12C^* mutations in advanced colorectal cancer.

#### 4.1.3. EGFR Inhibitors

*KRAS* mutation status is a critical factor predicting the efficacy of certain therapies [12,13,14]. These mutations lead to the constitutive activation of *KRAS*, which continuously triggers downstream signaling pathways independent of upstream receptor activation. As a result, the therapeutic efficacy of anti-EGFR agents, such as cetuximab, is significantly reduced in tumors harboring *KRAS* mutations. Cetuximab is a monoclonal antibody designed to inhibit EGFR by blocking ligand binding and subsequent receptor activation, thereby disrupting key signals involved in tumor growth [49]. Although some in vivo studies have demonstrated partial inhibition of tumor progression with cetuximab, further research is necessary to fully elucidate its clinical benefit, especially in the context of *KRAS*-mutant tumors [50].

#### 4.1.4. Src Pathway

Compared to other types of ovarian cancer, MOC shows the highest Src kinase activity, suggesting the potential use of targeted therapy against Src [51]. In vivo targeting Src with dasatinib combined with oxaliplatin has demonstrated significant antitumor effects in an MOC cell model in comparison to SOC [51]. The results of another study showed that inhibiting the Src pathway and tubulin polymerization (KX-01 inhibitor) significantly suppressed tumor growth in preclinical models of MOC. This effect was enhanced with the additional use of oxaliplatin [52].

#### 4.1.5. Farnesyltransferase Inhibitors (FTIs)

As mentioned above, MOC is characterized by a high frequency of *KRAS* mutations, reported in over 40% of cases [12,13,14]. The oncogenic activity of mutated *KRAS* relies on its correct localization to the inner surface of the plasma membrane, which is mediated by post-translational modification known as farnesylation [53]. This process is catalyzed by the enzyme farnesyltransferase and is essential for *KRAS* signaling through downstream pathways such as MAPK and PI3K/AKT, promoting tumor cell proliferation and survival. Farnesyltransferase inhibitors (FTIs), such as lonafarnib, were initially developed to disrupt Ras-driven oncogenesis by preventing the farnesylation of Ras proteins, thereby blocking their membrane localization and activity [53]. Although this approach is mechanistically sound, the clinical efficacy of FTIs in *KRAS*-mutant cancers, including MOCs, has been limited. One of the main reasons is that *KRAS* can undergo alternative prenylation by geranylgeranyltransferase, allowing it to maintain membrane association and signaling despite farnesyltransferase inhibition [54]. Some studies have tested FTIs on ovarian cancer cell lines (e.g., OVCAR-3), showing promising antitumor activity in preclinical models [55]. However, these studies do not specify mucinous ovarian carcinoma, which is a distinct and less common subtype. A phase II clinical trial involving five patients with mucinous ovarian carcinoma showed that adding lonafarnib to standard treatment with carboplatin and paclitaxel offered no additional benefit, as there were no observed improvements in progression-free survival or overall survival compared to chemotherapy alone [56]. It should also be noted that just one of five MOC patients was treated with the paclitaxel/carboplatin/lonafarnib combination. Nugawela et al. rightly pointed out that definitive conclusions regarding the efficacy of lonafarnib in the treatment of mucinous ovarian carcinoma cannot be drawn due to the absence of outcome analysis stratified by histological subtype [57]. Nevertheless, based on the overall findings of the trial, further investigation of lonafarnib in ovarian cancer lacks scientific justification. Thus, while the standalone efficacy of FTIs in MOCs remains limited, their role as part of combination therapies or in molecularly selected patients continues to be an area of active research.

### 4.2. Immunotherapy

#### 4.2.1. Checkpoint Inhibitors

It has been discovered that 14% of MOCs present a high level of PD-L1 protein, PD-L1 expression, and CD8+ T cell density, which could be a target for PD-L1 inhibitors such as Pembrolizumab or Atezolizumab [58]. However, in advanced stages of MOC, a higher concentration of PD-L1−TAMs (M2) and T-regulatory FOXP3+ cells points to a more immunosuppressive environment [58]. Atezolizumab is approved for treating various cancers, including non-small cell lung cancer, small cell lung cancer—used with chemotherapy for extensive-stage disease, triple-negative breast cancer (TNBC), urothelial carcinoma, hepatocellular carcinoma—combined with bevacizumab as a first-line treatment, and melanoma [59]. Given the PD-L1 expression in part of the patients with MOCs, researchers analyzed the role of Atezolizumab in these cases. It has been uncovered that the median progression-free survival did not improve significantly, and no evidence was shown to support adding PD-L1 inhibitors to the standard first-line treatment in patients with newly diagnosed ovarian cancer [60]. Pembrolizumab has been tested in recurrent ovarian cancer (ROC), following standard platinum-based CT. Patients exhibiting a high number of PD-L1-positive cells seemed to benefit the most from this treatment. Although the results were modest at best, it is probable that administering Pembrolizumab might be beneficial in patients with ROC after CT [61]. Meta-analysis of 15 clinical trials concerning the treatment of advanced ovarian cancer with PD-L1 inhibitors showed limited efficacy due to relatively low microsatellite instability and tumor mutational burden overall in EOCs. The strongest effects were achieved in combination with standard chemotherapy. Moreover, the addition of anti-VEGF resulted in a better response in patients treated with PD-L1 inhibitors [62]. Those factors limit the percentage of patients suitable for treatment with PD-L1 inhibitors for MOCs [63].

Liu et al. interestingly describes that targeting both cancer cells and their neighboring cells, such as immune and stromal cells in the tumor microenvironment, can improve cancer treatment by disrupting the supportive environment that tumors rely on, overcoming resistance, and enhancing the effectiveness of therapies through strategies like altering immune cell composition, blocking immunosuppressive cells, and reprogramming myeloid cells to fight tumors [64]. This concept is particularly applicable to mucinous ovarian carcinoma (MOC), which often exhibits limited immunogenicity but may still harbor exploitable immune checkpoints such as PD-L1. As discussed in the present review, mechanisms such as *KRAS* mutations and aberrant Wnt/β-catenin signaling may contribute to immune exclusion in MOCs, suggesting that rational combinations—for example, PD-L1 blockade with pathway inhibitors—may enhance responsiveness. Thus, the dual strategy could inform the development of next-generation immunotherapeutic regimens specifically tailored to the unique tumor-immune landscape of MOCs [64].

#### 4.2.2. Cancer Vaccines

A study on a lymph-node-targeted, *mKRAS*-specific amphiphile vaccine in pancreatic and colorectal cancer, both, similarly to MOCs, with high *KRAS* mutation rates, showed biomarker declines in 21 of 25 patients, including 6 with complete clearance. Ex vivo tests revealed a 58-fold increase in T-cell activity. A key advantage of these vaccines is their broad availability, given their set composition and no HLA restrictions [65].

Other notable examples of vaccines researched in OC are oregovomab, abagovomab, the p53-SLP vaccine, the DC vaccine loaded with tumor cell lysate, and CVac [66]. Both oregovomab and abagovomab target CA-125 and trigger an immune response, visible in a high specific T cell count. However, they did not significantly increase recurrence-free survival or overall survival. More studies are required to discover the best combination of these substances with other therapeutic agents [67,68,69].

#### 4.2.3. Adoptive T-Cell Therapy: CAR-T Cells

Cancer cells present antigens that are recognized by immune cells as foreign. This recognition triggers the activation of white blood cells, enabling them to locate and eliminate these abnormal cells. The participating cells include autologous T-cells, tumor-infiltrating lymphocytes, T-cell receptor-modified T-cells, chimeric antigen receptor T-cells (CAR-Ts), and NK cells. Adoptive T-cell therapy (ACT) is based on enhancing those physiological anti-cancer mechanisms. During the process, immune cells are extracted from the patient, then expanded and stimulated to later be reinfused, alongside growth factors such as interleukin-2 (IL-2) to boost their response [70,71]. While there are few studies analyzing the potential use of ACT specifically in MOC and its specific antigens, a meta-analysis of its role in ovarian cancers in patients with advanced or recurrent gynecologic cancer showed an overall objective response rate of 26.3%. Lymphodepletion has been observed to boost the efficacy of ACT [71].

#### 4.2.4. Hormonal Therapy

MOC rarely expresses estrogen receptors (ERs) and progesterone receptors (PRs). Retrospective studies generally report low levels of hormone receptor expression in MOC cases. A survey of 37 Chinese women found ER expression in seven cases and PR expression in nine cases [72]. Similarly, in Nigeria, a study of 24 cases of MOC shows the expression of ERs and PRs at 8.3% and 22.5%, respectively [73]. Another study involving 132 MOC cases identified ER expression in 14 cases (10.6%) [18]. In a survey of Mexican women with ovarian cancer, the number of MOC cases was too small to assess the hormonal profile. Among the nine MOC cases, only one showed PR expression, and three showed ER expression [74]. A study of 52 cases of MOC demonstrated hormone receptor expression limited to seromucinous tumors (11 cases, each expressing ER and PR) [75]. The presence of the ERs may be a target for therapy by using Letrozole. Response was observed in 17% of patients, while 26% showed no disease progression after 6 months of treatment. However, most patients had serous carcinoma. No cases of mucinous carcinoma were included [76].

#### 4.2.5. Synthetic Lethality Strategies

Synthetic lethality refers to a genetic interaction in which the simultaneous perturbation of two genes leads to cell death, whereas a defect in either gene alone is non-lethal [77,78]. In the context of cancer, this concept is exploited by targeting a gene that is dispensable for normal cells but becomes essential when a specific cancer-associated mutation is present [79]. Such an approach enables selective tumor cell killing: the cancer cell, harboring a particular mutation, dies when its synthetic lethal partner is inhibited, while normal cells (lacking the mutation) remain viable. This strategy effectively allows researchers to attack genetic alterations that were previously deemed “undruggable,” such as loss-of-function mutations in tumor suppressors or certain oncogene activations [80]. As a result, synthetic lethality has emerged as an important paradigm in precision oncology, offering a novel avenue to develop mutation-targeted therapies. Early successes validating this approach in clinical oncology underline its promise, although the field remains in its infancy, and continued research is expanding the roster of synthetic lethal targets [80].

MOC is a distinct subtype of ovarian cancer with a unique molecular profile that makes synthetic lethal strategies particularly compelling [81]. Unlike high-grade serous ovarian cancers (which often carry *TP53* or homologous recombination defects), MOC typically features recurrent alterations in cell signaling genes—for example, activating mutations in the RAS–MAPK pathway (*KRAS* and *BRAF*) or overexpression/amplification of receptor tyrosine kinases like HER2/*ERBB2*. These oncogenic alterations drive tumor growth but also create specific dependencies that can be therapeutically exploited. A synthetic lethal approach would involve identifying partner genes or pathways that MOC cells rely on due to their hallmark mutations and then pharmacologically targeting those partners. For instance, *KRAS*-driven tumors (a common scenario in MOC) have been shown to depend on certain cell-cycle regulators for survival, suggesting that inhibiting such a partner in *KRAS*-mutant MOCs could selectively eliminate the cancer cells [82]. By leveraging these mutation-specific vulnerabilities, synthetic lethality offers a promising framework to develop new therapies for MOCs that go beyond conventional chemotherapy and better address the biology of this challenging OC subtype.

#### 4.2.6. PARP Inhibitors

An example of a synthetic lethality strategy is the use of poly-ADP ribose polymerase (PARP) inhibitors in *BRCA*-mutated cancers. Several PARP inhibitors, such as niraparib, olaparib, and rucaparib, have been FDA-approved for ovarian cancer with *BRCA1/2* mutations. The National Comprehensive Cancer Network (NCCN) Clinical Practice Guidelines in Oncology do not include PARP inhibitors in the treatment of MOCs [83].

Due to the lack of *BRCA* mutations and low HR deficiency, MOC is unlikely to respond to PARP inhibition in monotherapy. However, combining PARP inhibitors with other inhibitors could induce a synthetic lethality effect in *BRCA*-proficient MOC [84]. It has been suggested that combining DNA methyltransferase (DNMT) inhibitors with PARP inhibitors can induce synthetic lethality in *BRCA*-proficient ovarian cancer cells by triggering epigenetic changes that disrupt HR, thereby mimicking *BRCA* mutations and increasing sensitivity to PARP inhibitors [85]. In another study, it was found that cells could be more sensitive to PARP inhibitors thanks to ATR inhibition. ATR inhibitor VE-821 sensitized OVCAR-8 cells to PARP inhibitor veliparib. OVCAR-8 is not a MOC cell line, but it is also *BRCA*-proficient [86]. ATR inhibitors also enhanced the therapeutic response to PARP inhibitors in ovarian cancer patient-derived xenograft (PDX) models [78]. Another potential therapeutic approach may be a PARP inhibitor, niraparib, in combination with a tyrosine kinase inhibitor, anlotinib, which showed promising antitumor activity in platinum-resistant OC patients [87,88].

MOCs may exhibit high PD-L1 expression [89]. Antagonistic PD-L1 has demonstrated a synergistic antitumor effect with PARP inhibitors [90]. Niraparib combined with the anti–PD-1 antibody pembrolizumab exhibited promising antitumor activity in recurrent platinum-resistant OC patients. This study included patients with various ovarian cancer subtypes, including MOCs, irrespective of *BRCA* mutations [91].

It was demonstrated that the knockdown of MRE11, an enzyme in the MRN complex, led to increased sensitivity to the PARP inhibitor BMN673 in clear-cell ovarian cancer (CCOC) cell lines. The MRN complex was negative in 9 out of 14 MOC samples, suggesting that MOC patients with a negative MRN complex may also benefit from PARP inhibitors [92]. Another promising combination is the PARP inhibitor, olaparib, with the cyclin-dependent kinases 4/6 (CDK4/6) inhibitor, palbociclib, which had synergistic effects against MYC-overexpressing ovarian cancer cells. Palbociclib induces HR deficiency through the downregulation of *MYC*-regulated genes, causing synthetic lethality with Olaparib [93]. This combination may be worth further investigation for MOC, as overexpression of *MYC* is frequently observed in this subtype [94].

#### 4.2.7. ATR Inhibitors

Ataxia telangiectasia and Rad3-related protein (ATR) inhibitors preferentially target HR-deficient tumor cells [95]. The majority of MOCs are HR-proficient; thus, the use of ATR inhibitors in MOC may be limited [18]. The potential ATR inhibitor target group might be MOC patients with AT-rich interactive domain-containing protein 1A (ARID1A)-mutated tumors. This mutation has been detected in 10,3% of MOC patients, and it has been shown that ATR inhibitors are synthetically lethal for tumors with this mutation [18,96]. ATR inhibitors are also synthetically lethal in *XRCC1*-deficient OC cells [97]. Additionally, ATR inhibitors may increase the therapeutic response for PARP-inhibitors, platinum-based chemotherapy, and topotecan [86,87].

#### 4.2.8. Other Synthetic Lethality Strategies

In MOC cell lines, genes in synthetic lethality with the PLK1 inhibitor, onvansertib, were identified. These genes are *JUND, CARD9*, and *BCL2L2* [98]. Additionally, strong synergism has been observed in MOC cell lines when PLK1 inhibitors, onvansertib and volasertib, were combined with paclitaxel and eribulin, as well as when they were paired with PIK75, a PI3K inhibitor [99]. Furthermore, the PLK1 inhibitors GSK317314A, GSK326090A, and BI2536 were synthetically lethal in the case of mismatch repair (MMR) deficiency in ovarian cancer. MOC patients had a higher incidence of MMR deficiency compared to serous ovarian cancer patients. Five out of eleven MOC samples were MMR-deficient, suggesting PLK1 inhibitors may be a potential therapeutic option for MOCs [100].

Another promising synthetic lethality strategy involves MEK inhibitor combinations. One of the MEK inhibitors, trametinib, combined with the PI3K inhibitor, buparlisib, shows promising antitumor activity in *KRAS*-mutant ovarian cancer patients [101]. Similarly, in *KRAS/NRAS* mutated type I epithelial ovarian cancers, including MOCs, the MEK inhibitor treatment resulted in tumor shrinkage, durable responses, and a CA125-related response [102]. Finally, the MEK inhibitor, pimasertib, combined with the dual PI3K/mTOR inhibitor, SAR245409, synergistically inhibited cell growth and induced apoptosis in MOC cell lines with *KRAS* mutations [103].

#### 4.2.9. Small Molecule Inhibitors for Wnt/β-Catenin Pathway and Other Deregulated Pathways

Database analyses show that Trefoil Factor 1 (TFF1) is predominantly overexpressed in MOCs compared to serous and other subtypes. Elevated TFF1 is linked to poor prognosis, increased cell viability and invasion, and cisplatin resistance, while its levels positively correlate with key oncogenes (*CCND1*, *c-myc*, and *Twist*) and the aberrant activation of the Wnt/β-catenin pathway [104]. Similarly, inorganic pyrophosphatase (PPA1) is markedly upregulated in epithelial ovarian tumors. Higher PPA1 levels correlate with advanced grades and stages and poorer survival outcomes. Functional studies indicate that reducing PPA1 impairs cell migration, epithelial–mesenchymal transition (EMT), and metastasis, likely via enhanced dephosphorylation and nuclear translocation of β-catenin, suggesting its potential as a therapeutic target [105]. Dickkopf-related protein 1 (DKK1) exhibits a dual role. In vitro, its suppression enhances Wnt signaling; however, in vivo, high DKK1 levels are associated with larger tumors and reduced immune cell infiltration. This implies that DKK1 may promote tumor growth by modulating the tumor microenvironment, making it a promising target to counteract chemoresistance [106,107].

Ten-eleven translocation 1 (TET_1_) appears to act as a tumor suppressor in EOCs, with lower levels found in advanced stages. Overexpression of TET_1_ increases the Wnt antagonists SFRP2 and DKK1, thereby deactivating the Wnt/β-catenin pathway, reducing cell migration, and reversing EMT. Mouse models further confirm that TET_1_ overexpression inhibits tumor growth [108].

**Figure 2:**
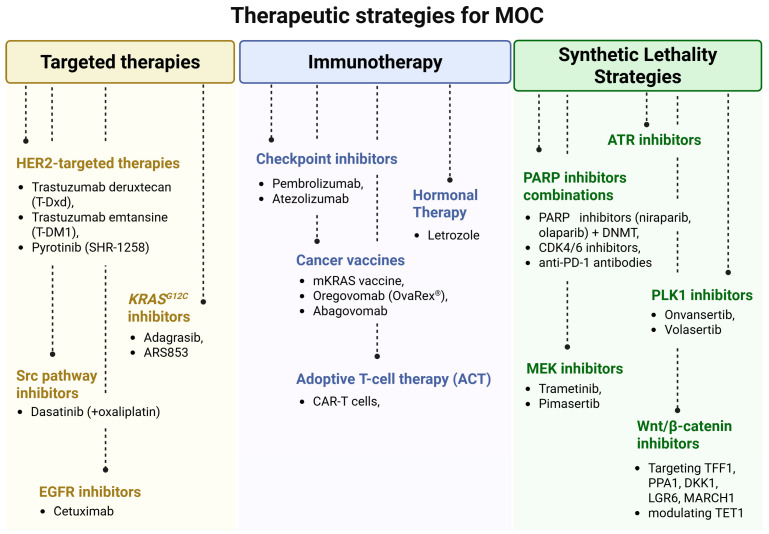
Therapeutic strategies of MOC. Created in BioRender. Łapińska, Z. (2025) https://BioRender.com/m9x5yi6.

Leucine-rich repeat-containing G protein-coupled receptor 6 (LGR6) is upregulated in ovarian cancer—including mucinous adenocarcinomas—and is linked to poor chemotherapeutic response and reduced progression-free survival. Downregulation of LGR6 diminishes the cancer stem cell-like phenotype and chemoresistance, re-sensitizing cells to chemotherapy [109]. Finally, membrane-associated RING-CH 1 (MARCH1) is significantly overexpressed in ovarian cancer tissues relative to non-tumorous samples. Knocking down MARCH1 in SKOV3 cells reduces proliferation, invasion, and migration by attenuating NF-κB and Wnt/β-catenin signaling, underscoring its role as an oncogenic driver and a promising therapeutic target [110]. Collectively, these studies highlight critical regulators of the Wnt/β-catenin and related pathways in ovarian mucinous carcinoma. Targeting TFF1, PPA1, DKK1, LGR6, and MARCH1—while considering the tumor-suppressive role of TET_1_—may offer novel therapeutic avenues to overcome chemoresistance and improve patient outcomes. The described therapeutic strategies have been summarized in Figure 2.

## 5. Summary and Conclusions

MOC is a rare subtype of ovarian cancer, accounting for only about 3–10% of all ovarian cancers [2]. Its rarity makes it difficult to conduct large-scale studies and clinical trials with a sufficient number of patients to draw robust conclusions. In many studies, only a few patients diagnosed with MOC are included among a much larger number of participants with other ovarian cancer subtypes. As a result, treatment strategies are often extrapolated from studies on other ovarian cancer subtypes, which may not be as effective. Another challenge is the heterogeneity, as it can exhibit diverse biological and molecular characteristics. Additionally, MOC can be difficult to distinguish from other types of ovarian tumors, further complicating research. Moreover, this OC subtype often shows resistance to standard chemotherapy regimens commonly used.

It should be noted that mucinous ovarian cancer offers a valuable model for designing next-generation treatment regimens that optimally balance cell-autonomous and microenvironmental interventions. MOC’s unique tumor biology, including its distinct molecular alterations and interactions with the immune microenvironment, underscores the need for therapeutic strategies that go beyond targeting cancer cells alone. As highlighted by Joshi et al., the future of cancer treatment is moving toward personalized and precision approaches that integrate molecular profiling with interventions aimed at modulating the tumor immune microenvironment [111]. These emerging strategies—such as cell and gene therapies, immune checkpoint inhibitors, and combination regimens—are designed to restore the disrupted cancer-immune balance and overcome resistance mechanisms that often limit the efficacy of monotherapies. By leveraging deeper insights into both the intrinsic properties of tumor cells and the complex crosstalk within the tumor microenvironment, new therapies hold promise for more durable and effective responses in MOCs and other cancers, paving the way for individualized treatment regimens that address both cell-intrinsic and extrinsic drivers of disease progression [112].

Nevertheless, more research is needed to find effective treatment options for MOCs. Studies should focus specifically on patients diagnosed with this OC subtype and be conducted on a large group. Table 1 below provides an overview of therapeutic approaches, including their molecular targets, representative agents, current clinical evidence, and associated comments or limitations.

Debulking is vital for achieving maximal cytoreduction and improving overall survival for patients with MOC. Given the low efficacy of standard chemotherapy in advanced MOC, novel approaches, such as molecular-based therapy matching, are essential. Promising targeted treatments include HER2-directed agents (e.g., ADCs) and inhibitors of KRAS, Src, and EGFR [36,47,50,51,52]. Immunotherapy, particularly when combined with standard regimens, may offer additional benefits. Although ER/PR expression is rare in MOC, these receptors remain potential targets. Furthermore, synthetic lethality strategies—such as combining PARP inhibitors with other agents—and targeting aberrant Wnt/β-catenin signaling could help overcome chemoresistance. Continued research is crucial to developing effective therapies for MOC.

## Figures and Tables

**Table 1:** Summary of therapeutic strategies for mucinous ovarian carcinoma (MOC).

Therapeutic Strategy	Mechanism/Targets	Therapies/Agents	Clinical Evidence/Trials	Remarks and Limitations
**Standard therapies**				
Surgery	Cytoreduction	Debulking surgery, fertility-sparing surgery	Established clinical practice (e.g., NCT05123807)	Critical for staging; controversial role of lymphadenectomy and appendectomy.
Chemotherapy	DNA damage	Platinum (cisplatin) + Taxanes (paclitaxel)	Standard practice; resistance is common	Frequent multidrug resistance (MDR) due to P-gp, MRP, and low *BRCA* mutation rate
**Targeted therapies**				
HER2-targeted therapies	HER2 overexpression	Trastuzumab deruxtecan, trastuzumab emtansine, pyrotinib	Clinical trials ongoing; promising initial results in HER2+ cases (NCT04482309, NCT04639219)	Particularly effective for HER2-overexpressing tumors; resistance may develop.
*KRAS^G12C^* inhibitors	*KRAS* mutations	Adagrasib, ARS853	Early clinical trials (e.g., KRYSTAL-10); no specific trials yet for MOC	Promising in *KRAS^G12C^* mutant cancers; further MOC studies needed.
Src pathway inhibitors	Src kinase activity	Dasatinib (+oxaliplatin)	Preclinical studies	High activity observed; limited clinical evidence
EGFR inhibitors	EGFR signaling	Cetuximab	Limited in vivo studies	Mixed results; further clinical validation required.
**Immunotherapy**				
Checkpoint inhibitors	PD-L1 expression	Pembrolizumab, Atezolizumab	Modest clinical response; limited benefit in MOC	Best results in combination therapy due to immunosuppressive environment.
Cancer vaccines	*KRAS*-specific immunity	*mKRAS* vaccine, oregovomab, abagovomab	Limited clinical efficacy; early trials promising in related cancers	Vaccine development promising due to high *KRAS* mutation rates.
Adoptive T-cell therapy (ACT)	Antigen-specific immune response	CAR-T cells	Limited ovarian cancer data; promising results with lymphodepletion	General evidence encouraging; requires MOC-specific antigens.
Hormonal Therapy	ER/PR receptor expression	Letrozole	Limited applicability due to low ER/PR expression	Rarely effective due to infrequent hormone receptor positivity in MOC.
**Synthetic Lethality Strategies**				
PARP inhibitor combinations	DNA repair pathways	PARP inhibitors (niraparib, olaparib) + DNMT, ATR inhibitors, CDK4/6 inhibitors, anti-PD-1 antibodies	Preclinical studies; clinical trials required	Monotherapy is limited due to HR proficiency; combinations are promising.
ATR inhibitors	HR deficiency	ATR inhibitors	Synthetic lethality in ARID1A/XRCC1 mutations	Potential for specific genetic profiles in MOC.
PLK1 inhibitors	Cell cycle regulation	Onvansertib, Volasertib	Synthetic lethality with MMR deficiency; preclinical studies encouraging	Effective in MMR-deficient subgroups; clinical evidence needed
MEK inhibitors	KRAS/NRAS pathway	Trametinib, Pimasertib	Effective in combination with PI3K/mTOR inhibitors in preclinical studies	Encouraging results; applicable in *KRAS*-mutant MOC.
Wnt/β-catenin inhibitors	Pathway deregulation	Targeting TFF1, PPA1, DKK1, LGR6, MARCH1; modulating TET1	Preclinical evidence; significant association with chemoresistance	Potentially valuable in overcoming resistance, but further clinical validation is required.

## Data Availability

No new data were created or analyzed in this study.

## Abbreviations

The following abbreviations are used in this manuscript:

MOC	Mucinous Ovarian Carcinoma
OC	Ovarian Cancer
SOC	Serous Ovarian Carcinoma
EOC	Epithelial Ovarian Cancer
HGSOC	High-Grade Serous Ovarian Cancer
CCOCs	Clear-Cell Ovarian Cancers
ROC	Recurrent Ovarian Cancer
TNBC	Triple-Negative Breast Cancer
ERs	Estrogen Receptors
PRs	Progesterone Receptors
CT	Chemotherapy
HR	Homologous Recombination
MMR	Mismatch Repair
HIPEC	Hyperthermic Intraperitoneal Chemotherapy
MDR	Multidrug Resistance
MRP	Multidrug Resistance-Associated Protein
G6PD	Glucose-6-Phosphate Dehydrogenase
P-gp	P-Glycoprotein
ADCs	Antibody–Drug Conjugates
T-DXd	Trastuzumab Deruxtecan
NK cells	Natural Killer Cells
CAR-Ts	Chimeric Antigen Receptor T-Cells
ACT	Adoptive T-Cell Therapy
IL-2	Interleukin-2
PARP	Poly-ADP Ribose Polymerase
NCCN	National Comprehensive Cancer Network
DNMT	DNA Methyltransferase
PDXs	Patient-Derived Xenografts
CDK4/6	Cyclin-Dependent Kinases 4/6
ATR	Ataxia Telangiectasia and Rad3-Related Protein
ARID1A	AT-Rich Interactive Domain-Containing Protein 1A
XRCC1	X-ray Repair Cross Complementing 1
PLK1	Polo-Like Kinase 1
TFF1	Trefoil Factor 1
PPA1	Pyrophosphatase 1
EMT	Epithelial–Mesenchymal Transition
DKK1	Dickkopf-Related Protein 1
TET_1_	Ten-Eleven Translocation 1
LGR6	Leucine-Rich Repeat-Containing G Protein-Coupled Receptor 6
MARCH1	Membrane-Associated RING-CH 1
